# Dysregulated Urinary Extracellular Vesicle Small RNAs in Diabetic Nephropathy: Implications for Diagnosis and Therapy

**DOI:** 10.1210/jendso/bvae114

**Published:** 2024-06-12

**Authors:** Hamad Ali, Md Zubbair Malik, Mohamed Abu-Farha, Jehad Abubaker, Preethi Cherian, Irina Al-Khairi, Rasheeba Nizam, Sindhu Jacob, Yousif Bahbahani, Abdulnabi Al Attar, Thangavel Alphonse Thanaraj, Fahd Al-Mulla

**Affiliations:** Department of Medical Laboratory Sciences, Faculty of Allied Health Sciences, Health Sciences Center (HSC), Kuwait University, Jabriya, PO Box 24923, Safat 13110, Kuwait; Department of Genetics and Bioinformatics, Dasman Diabetes Institute (DDI), PO Box 1180, Dasman 15462, Kuwait; Division of Nephrology, Mubarak Al-Kabeer Hospital, Ministry of Health, Jabriya, PO Box 24923, Safat 13110, Kuwait; Department of Genetics and Bioinformatics, Dasman Diabetes Institute (DDI), PO Box 1180, Dasman 15462, Kuwait; Department of Biochemistry and Molecular Biology, Dasman Diabetes Institute (DDI), PO Box 1180, Dasman 15462, Kuwait; Department of Translational Medicine, Dasman Diabetes Institute (DDI), PO Box 1180, Dasman 15462, Kuwait; Department of Biochemistry and Molecular Biology, Dasman Diabetes Institute (DDI), PO Box 1180, Dasman 15462, Kuwait; Department of Biochemistry and Molecular Biology, Dasman Diabetes Institute (DDI), PO Box 1180, Dasman 15462, Kuwait; Department of Biochemistry and Molecular Biology, Dasman Diabetes Institute (DDI), PO Box 1180, Dasman 15462, Kuwait; Department of Genetics and Bioinformatics, Dasman Diabetes Institute (DDI), PO Box 1180, Dasman 15462, Kuwait; Department of Genetics and Bioinformatics, Dasman Diabetes Institute (DDI), PO Box 1180, Dasman 15462, Kuwait; Division of Nephrology, Mubarak Al-Kabeer Hospital, Ministry of Health, Jabriya, PO Box 24923, Safat 13110, Kuwait; Medical Division, Dasman Diabetes Institute (DDI), PO Box 1180, Dasman 15462, Kuwait; Medical Division, Dasman Diabetes Institute (DDI), PO Box 1180, Dasman 15462, Kuwait; Department of Genetics and Bioinformatics, Dasman Diabetes Institute (DDI), PO Box 1180, Dasman 15462, Kuwait; Department of Translational Medicine, Dasman Diabetes Institute (DDI), PO Box 1180, Dasman 15462, Kuwait

**Keywords:** diabetic nephropathy, PTEN, miR-151a-3p, miR-182-5p, T2D, urinary extracellular vesicles, SMAD4, VEGFA, miRNA, exosome

## Abstract

**Background:**

Diabetic nephropathy (DN) represents a major chronic kidney disorder and a leading cause of end-stage renal disease (ESRD). Small RNAs have been showing great promise as diagnostic markers as well as drug targets. Identifying dysregulated micro RNAs (miRNAs) could help in identifying disease biomarkers and investigation of downstream interactions, shedding light on the molecular pathophysiology of DN. In this study, we analyzed small RNAs within human urinary extracellular vesicles (ECVs) from DN patients using small RNA next-generation sequencing.

**Method:**

In this cross-sectional study, urine samples were collected from 88 participants who were divided into 3 groups: type 2 diabetes (T2D) with DN (T2D + DN, n = 20), T2D without DN (T2D − DN, n = 40), and healthy individuals (n = 28). The study focused on isolating urinary ECVs to extract and sequence small RNAs. Differentially expressed small RNAs were identified, and a functional enrichment analysis was conducted.

**Results:**

The study revealed a distinct subset of 13 miRNAs and 10 Piwi-interacting RNAs that were significantly dysregulated in urinary ECVs of the DN group when compared to other groups. Notably, miR-151a-3p and miR-182-5p exhibited a unique expression pattern, being downregulated in the T2D − DN group, and upregulated in the T2D + DN group, thus demonstrating their effectiveness in distinguishing patients between the 2 groups. Eight driver genes were identified *PTEN*, *SMAD2*, *SMAD4*, *VEGFA*, *CCND2*, *CDK6*, *LIN28B*, and *CHD1*.

**Conclusion:**

Our findings contribute valuable insights into the pathogenesis of DN, uncovering novel biomarkers and identifying potential therapeutic targets that may aid in managing and potentially decelerating the progression of the disease.

Diabetic nephropathy (DN) is a progressive chronic kidney complication that affects approximately 30% of patients with type 1 diabetes (T1D) and 40% of individuals with type 2 diabetes (T2D). It is regarded as the leading cause of end-stage renal disease (ESRD) worldwide [1, 2]. The prevalence of DN has increased in recent years in proportion to the rising prevalence of diabetes, with DN emerging as a major contributor to morbidity and mortality in this patient population [3]. The clinical diagnosis of DN relies on persistent urine proteinuria and progressive reduction in the estimated glomerular filtration rate (eGFR) [4]. However, diagnostic limitations persist as challenges for DN management. Kidney damage can occur even before urinary albumin is detected [5]. In addition, reduction in kidney function is not always correlated with increased albuminuria [6]. Current diagnostic and therapeutic approaches for DN are not highly effective in reducing ESRD rates. Therefore, there is an urgent need for earlier and more sensitive diagnostic markers as well as novel drug targets that can be utilized for new therapeutic approaches for DN [7].

In recent years, interest in exosomes has been on the rise with potential clinical benefits. Exosomes, extracellular vesicles (ECVs) with a diameter ranging from 40 to 160 nm, have been shown to contain a variety of components, including several types of nucleic acids (such as miRNAs and Piwi-interacting RNAs [piRNAs]), proteins, lipids, amino acids, and other metabolites [8]. Initially considered as byproducts of cell damage or homeostasis, recent advancements have highlighted the crucial role of exosomes in facilitating intercellular communication [9]. They actively govern essential cellular processes, such as signal transduction and immune response [8]. Exosomes participate in cell-cell communication within the nephron, influencing both renal and pathophysiological processes [10]. Their involvement has been demonstrated in various renal diseases and disorders, including DN [11]. Several reports have suggested that the exosomal contents, specifically small noncoding RNAs such as miRNAs, play a role in DN pathogenesis by influencing multiple signaling pathways and cellular processes, including inflammation and autophagy [11]. In addition, it has been shown that the molecular signatures of urinary ECVs contents, including proteins and small noncoding RNAs, undergo alternation in response to DN pathogenesis [12-15]. These altered profiles of urinary exosomes in DN may serve as promising biomarkers for the diagnosis, prognosis, and monitoring of diseases along with other biomarkers recently identified [16-19]. In addition, they can offer valuable insights into the potential development of therapies.

In this study, we performed a global analysis of the small RNA content of urinary ECVs from patients with type 2 diabetes and nephropathy (T2D + DN) and compared them with those from individuals with type 2 diabetes without nephropathy (T2D − DN) and healthy subjects. We identified miRNAs significantly dysregulated in DN patients and further analyzed the network of common target mRNAs affected by these miRNAs. Our findings point to potential novel biomarkers for DN, reveal possible therapeutic targets for the disease, and enhance our understanding of DN pathophysiology.

## Materials and Methods

### Patient Recruitment

A total of 88 subjects were recruited into the study and were allocated to 3 groups: T2D + DN (n = 20), T2D − DN (n = 40), and healthy individuals (n = 28). Patients were recruited from the Dasman Diabetes Institute. This study was reviewed and approved by the Ethical Review Committee at Dasman Diabetes Institute (reference: RA/121/2019). Individuals eligible for participation were required to be adults aged 18 years or older and capable of providing informed consent. The inclusion criteria necessitated a verified clinical diagnosis of DN and T2D. The clinical diagnosis of T2D relied on the presence of persistent hyperglycemia (fasting glucose level > 7 mmol/L and 2-hour fasting blood glucose > 11 mmol/L) and normal kidney function. Individuals with DN received clinical diagnosis from a nephrologist based on the American Diabetes Association criteria [20]. These patients exhibited marked T2D, along with a sustained increase in albumin to creatinine ratio (ACR) > 30 mg/g and/or a persistent decline in eGFR (< 60 mL/min per 1.73 m^2^). Healthy individuals without any reported diagnosis of T2D or kidney complications, and with clinical parameters within normal ranges, were recruited from the Kuwait Adult Diabetes Epidemiological Multidisciplinary (KADEM) study. Patients with nondiabetic kidney disease, heart failure, active infection, acute/chronic inflammatory disease, allergic conditions, autoimmune diseases, malignancies, and T1D were excluded.

### Urine Collection and ECVs Purification

Urine samples were collected in urine collection and preservation tubes (Norgen Biotech Corp.18111) as previously described [21]. Briefly, the sample underwent a series of centrifugation and concentration steps. The resulting pellet was resuspended in 500 µL of PBS and stored at 40 °C.

### Total RNA Extraction From Urinary ECVs

ECVs total RNA was extracted using Urine Exosome Purification and RNA Isolation Maxi Kit, Norgen, Canada (Cat. 58800) according to the manufacturer's protocol.

### Small RNA Library Preparation and Sequencing

We followed the methods described in detail in [21]. Library preparation was performed using QIAseq miRNA Library Kit (331502, Qiagen, Hilden, Germany) following the manufacturers recommendations. Each run used 10 ng of purified small RNA as startup material. The procedure involves consecutively ligating 3′ and 5′ end adapters, followed by universal cDNA synthesis with unique molecular index assignment. The synthesized cDNA libraries underwent further amplification through PCR cycles and purification using Qiagen QMN beads. The final libraries were prepared, validated, and quantified using the bioanalyzer (Agilent, California, USA) and qubit fluorometer (Thermofisher Scientific, Massachusetts, USA), respectively. Sequencing was conducted on the MiSeq system using the Miseq 150 cycle version 3 kit (MS-102-3001, Illumina Inc., USA). The resulting Fastq files were analyzed using Gene Globe data analysis webtools. Normalization was performed using the Trimmed Mean of M (edgeR) method, calculating a linear scaling factor for each sample based on a weighted mean after reducing the dataset by log fold-changes relative to control samples and absolute intensity [22]. Candidate piRNA and miRNAs were selected based on the criteria of log2 fold change > 2 and a *P* value < .05.

### Identification of Differentially Expressed Small RNA and Functional Enrichment Analysis of Key miRNAs

The confirmed interactions between miRNAs and their target mRNAs were obtained using miRNet (https://www.mirnet.ca/), an interactive web tool. This tool retrieves data on miRNA-target interactions, combining both computationally predicted, and experimentally validated information from miRTarBase [23], miRDB [24], and TargetScan [25] databases. We identified the miRNAs from the study in these databases, considering them as candidate miRNAs in conjunction with the mRNAs [26]. The significantly correlated pairs of these miRNA-mRNA interactions were employed to construct a co-expression network using Cytoscape 3.6.1. The cytoHubba v.0.1 plug-in of Cytoscape was used to select potential hub genes from the identified differentially expressed miRNAs (DE-miRNAs) [27, 28]. The edge percolated component (EPC) centrality analysis of the miRNA-mRNA regulatory network was used to determine the driver genes targeted by the key miRNA [29, 30]. The significant biological pathways associated with the identified candidate miRNAs were determined using the DIANA-microT web server [31]. The most statistically enriched GO terms were generated using the ggplot2 visualization package [32].

### Statistical Analysis

The shortlisted markers were observed to be significant after Bonferroni correction for multiple comparisons (*P* < .01). We generated receiver operating characteristic (ROC) curves, which assess the trade-off between sensitivity and specificity in predicting a dichotomous outcome across various values. The area under the ROC curve (AUC) serves as an additional indicator of test performance. AUC, CIs, and *P* values for all ROC curves, as well as individual ROC curves for each piRNA and miRNA, were computed. These results were presented alongside their respective sensitivity and specificity values. The heatmap was generated using SRPLOT (available at https://www.bioinformatics.com.cn).

## Results

The study included 88 patients: 35 male (40%) and 53 female (60%). Table 1 shows the basic clinical and pathological characteristics of each group.

**Table 1.** Basic clinical and pathological characteristics

Characteristic	Healthy controls (n = 28)	Type 2 diabetes patients without nephropathy (n = 40)	Type 2 diabetes patients with nephropathy (n = 20)	*P* value (all 3 groups)	*P* value (DN vs non-DN)
Age at visit, y	49.9 (8.5)	62.4 (9.1)	63.4 (9.3)	<.001	.702
BMI, kg/m^2^	31.9 (7.4)	34.1 (8.1)	33.4 (5.4)	.524	.765
Glucose, mmol/L	5.1 (0.6)	9.4 (3.3)	9.3 (3.9)	<.001	.951
HbA1c, %	5.5 (0.7)	7.2 (1.5)	7.5 (1.3)	<.001	.417
ACR	8.7 (11.6)	14 (7.7)	706.3 (7.7)^$^	<.001	<.001
eGFR, mL/min/1.73 m^2^	102 (14.3)	81.06 (21.2)	71 (31.5)	<.001	.115
BUN, mmol/L	4.6 (1.2)	5.7 (2.34)	11.8 (20.2)	.040	.068
Creatinine, µmol/L	70.5 (16.3)	68.2 (27.1)	100.7 (40.3)	.120	.002
Albumin, g/L	38.5 (3.2)	35.4 (6.8)	37.4 (3.1)	.071	.234
HDL, mmol/L	1.4 (0.3)	1.3 (0.4)	1.04 (0.3)	.009	.010
LDL, mmol/L	3.4 (0.9)	1.8 (1.1)	1.6 (0.7)	<.001	.470

Data are presented as mean and SD and ^$^median.

Abbreviations: ACR, urine albumin to creatinine ratio; BMI, body mass index; BUN, blood urea nitrogen; eGFR, estimated glomerular filtration rate, HbA1c, glycated hemoglobin; HDL, high-density lipoprotein, LDL, low-density lipoprotein.

### Expression Profiles of Urinary ECV miRNAs Isolated From T2D − DN and T2D + DN Patients

**Figure 1.**
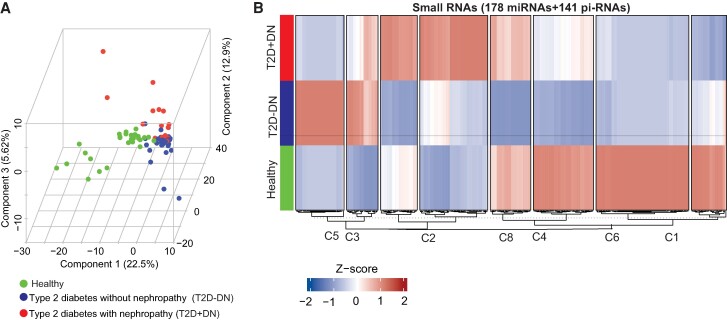
microRNA sequencing (miRNAseq) identifies differential expression of microRNAs (DE-miRNAs) from urinary exosomes from healthy individuals, patients with type 2 diabetes without nephropathy (T2D − DN), and those with type 2 diabetes with nephropathy (T2D + DN). (A) Principal component analysis of the miRNA expression data from healthy, T2D − DN, and T2D + DN. (B) Heatmap of the differentially expressed miRNA in healthy, T2D − DN, and T2D + DN. The red boxes indicate higher expression and blue boxes indicate the lower expression miRNAs.

Characterization of the purified urinary ECV based on size using scanning electron microscope showed a diameter in the range of 40 to 150 nm (Supplementary Fig. S1 [33]). The expression profiles of urinary ECVs miRNAs and piRNAs from T2D − DN patients were compared with the expression profiles from the nondiabetic individuals. Similarly, the expression profiles of urinary ECVs miRNAs and piRNAs from the 20 T2D + DN patients were compared with nondiabetic individuals. Principal component analysis of the miRNA expression data demonstrated clustering of T2D − DN, T2D + DN patients, and of controls with a separation between the 2 clusters (Fig. 1A). The results of heatmap clustering based on the expression pattern of all 178 detectable miRNAs and 141 piRNAs of T2D − DN, T2D + DN, and control groups are shown in Fig. 1B. Transcript cluster heatmaps indicated that the optimal number of clusters was 8; the larger cluster (n = 71 miRNAs & piRNA) was designated C6 and the smaller cluster (n = 24 miRNAs & piRNA) was designated C3 (Supplementary Table S1 [34]). Clusters C3 and C5 revealed the same changes in expression of miRNA/piRNAs in healthy and T2D + DN but higher expression in T2D − DN. Clusters C2 and C8 revealed higher expression of miRNA/piRNAs in T2D + DN patients than T2D − DN patients, whereas clusters C1, C6, and C4 revealed lower expression of miRNA/piRNAs in T2D − DN and T2D + DN patients (Supplementary Table S1 [34]). Cluster C7 revealed the same changes in the higher expression of miRNA/piRNAs in healthy and T2D + DN but lower expression in T2D − DN.

**Figure 2.**
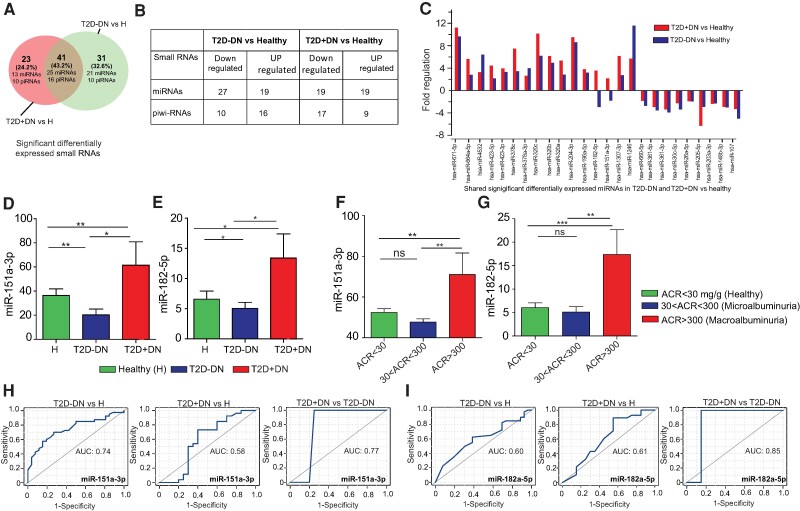
Expression pattern of dysregulated miRNAs in type 2 diabetes without nephropathy (T2D − DN) and type 2 diabetes with nephropathy (T2D + DN) urinary exosomes: (A) Venn diagram shows intersections the significant differentially expressed miRNA (DE-miRNA) among the T2D − DN and T2D + DN. (B) Table shows regulation of small RNA (sRNA) among T2D − DN and T2D + DN. (C) Fold change of shared differentially expressed miRNAs in T2D − DN and T2D + DN with comparison to healthy individuals. (D-E) Expression of miR-151a-3p and miR-182-3p by signal values between healthy, T2D − DN, and T2D + DN patients. (F-G) Box plot shows the expression of prognostic markers (miR-151a3p and miR-182-5p) by signal values between T2D + DN and T2D − DN patients with normal individual (ACR < 30 mg/g), microalbuminuria (30 < ACR < 300), and macroalbuminuria (ACR > 300 mg/g). (H-I) ROC curves for miR-151a-3p and miR182-5p of T2D − DN vs heathy, T2D + DN vs healthy and T2D + DN vs T2D − DN. The area under the ROC curve (AUC) represents the accuracy of the miR-151a-3p and miR-182-5p prognostic biomarkers in T2D − DN and T2D + DN. *indicates *P* < .05, **indicates *P* < .01, ***indicates *P* < .001, and “ns” indicates not significant.

Differential expression analysis revealed that 46 miRNAs (27 downregulated and 19 upregulated) were significantly differentially expressed between the controls and T2D − DN patients with *P* < .05 (Fig. 2A and 2B). We also observed that 38 miRNAs (19 downregulated and 19 upregulated) were significantly differentially expressed between controls and T2D + DN patients. The 21 significant DE-miRNAs were expressed in T2D − DN (Fig. 2A, Table 2) and 13 DE-miRNAs expressed in T2D + DN (Fig. 2A, Table 3). Out of 319 small miRNAs, we found 25 miRNAs (Fig. 2A and 2C) and 16 piRNAs (Supplementary Table S2 [34]) were significantly differentially expressed in T2D − DN as well as T2D + DN patients. We also observed 10 DE-piRNAs were expressed in T2D − DN (Supplementary Table S3 [34]) and 10 DE-piRNAs expressed in T2D + DN (Supplementary Table S4 [34]). Of these 25 shared miRNAs, we found 11 miRNAs in T2D − DN patients and 9 miRNAs in T2D + DN were downregulated (Fig. 2C, Table 4). Similarly, we also found 14 miRNAs in T2D − DN patients and 16 miRNAs in T2D + DN were upregulated (Fig. 2C, Table 4).

**Table 2.** Significant differentially expressed miRNAs in type 2 diabetes without nephropathy (T2D − DN) vs healthy

S. No.	miRNA ID	Fold regulation (T2D − DN vs healthy)	*P* value
1.	hsa-miR-9-5p	−2.27	.024832
2.	hsa-miR-92a-3p	−2.13	.009792
3.	hsa-miR-891a-5p	−3.93	.020414
4.	hsa-miR-744-5p	−3.33	.011081
5.	hsa-miR-532-5p	−1.84	.036956
6.	hsa-miR-363-3p	−4.54	.00031
7.	hsa-miR-338-3p	−2.58	.044152
8.	hsa-miR-30e-3p	−2.05	.017425
9.	hsa-miR-26b-5p	−1.7	.04021
10.	hsa-miR-204-5p	−1.86	.014252
11.	hsa-miR-16-5p	−1.67	.047319
12.	hsa-miR-146b-5p	−3.06	.026059
13.	hsa-miR-141-3p	−1.74	.0322
14.	hsa-miR-125b-5p	−1.69	.044593
15.	hsa-miR-125a-5p	−2.11	.00428
16.	hsa-miR-10a-3p	−3.24	.014664
17.	hsa-miR-8485	4.95	.009048
18.	hsa-miR-7704	4.29	.002462
19.	hsa-miR-6087	5.67	.00017
20.	hsa-miR-4516	6.75	8.21E−09
21.	hsa-miR-3656	7.91	4.33E−13

**Table 3.** Significant differentially expressed urinary extracellular vesicular miRNAs in type 2 diabetes with nephropathy (T2D + DN) vs healthy

S. No.	Differentially expressed miRNA	Fold change (T2D + DN vs healthy)	*P* value
1.	hsa-miR-3960	−7.2	.0043286
2.	hsa-miR-30b-5p	−3.7	.0286945
3.	hsa-miR-29c-3p	−2.18	.0146151
4.	hsa-miR-29a-3p	−2.91	.0013709
5.	hsa-miR-27a-3p	−4.36	.0026631
6.	hsa-miR-23b-3p	−4.16	2.528E−05
7.	hsa-miR-23a-3p	−5.02	4.822E−05
8.	hsa-miR-223-3p	−5.48	.0063404
9.	hsa-miR-142-5p	−8.39	.0001108
10.	hsa-miR-128-3p	−4.11	.0065145
11.	hsa-miR-874-3p	2.87	.0466693
12.	hsa-miR-199b-3p	7.65	.0001769
13.	hsa-miR-375	3.15	.0079878

**Table 4.** Significant shared differentially expressed urinary extracellular vesicular miRNAs in type 2 diabetes without nephropathy (T2D − DN) vs healthy and type 2 diabetes with nephropathy (T2D + DN) vs healthy

		(T2D − DN vs healthy)		(T2D + DN vs healthy)	
S. No.	DE-miRNA	Fold change	*P* value	Fold change	*P* value
1.	hsa-miR-107	−5.02	.000007	−3.31	.006926
2.	hsa-miR-148b-3p	−3.01	.001775	−2.91	.015193
3.	hsa-miR-203a-3p	−2.3	.004299	−2.37	.021347
4.	hsa-miR-205-5p	−2.9	.000167	−6.32	.00000009
5.	hsa-miR-26a-5p	−1.95	.013386	−1.92	.042271
6.	hsa-miR-30c-5p	−3.39	.001962	−2.26	.000437
7.	hsa-miR-361-3p	−3.92	.001452	−3.37	.021368
8.	hsa-miR-361-5p	−3.56	.005946	−2.95	.01126
9.	hsa-miR-660-5p	−2.75	.045127	−1.83	.009273
10.	hsa-miR-1246	11.6	.0000004	5.69	.000218
11.	hsa-miR-1307-3p	2.71	.000979	6.15	.0000001
12.	hsa-miR-151a-3p	−1.8	.0026164	2.17	.0011401
13.	hsa-miR-182-5p	−2.96	.000663	3.53	.000516
14.	hsa-miR-196a-5p	3.2	.000931	3.79	.011608
15.	hsa-miR-204-3p	8.63	.010186	9.51	.011607
16.	hsa-miR-320a	2.84	.000116	5.34	.0000005
17.	hsa-miR-320b	4.94	.00002	6.14	.000538
18.	hsa-miR-320c	6.19	.0000067	10.14	.00002
19.	hsa-miR-378a-3p	3.97	.0000029	2.61	.002763
20.	hsa-miR-378c	3.44	.0000035	7.46	.007745
21.	hsa-miR-423-3p	3.29	.003931	3.93	.00347
22.	hsa-miR-423-5p	2.15	.003986	4.44	.000001
23.	hsa-miR-4532	6.39	.000175	3.28	.02217
24.	hsa-miR-664a-5p	2.78	.032326	5.61	.001117
25.	hsa-miR-671-5p	9.64	.0000008	11.24	.00000017

### Dysregulated miRNAs in T2D − DN and T2D + DN Urinary Exosomes

The heatmap clustering of 25 common dysregulated DE-miRNAs in T2D − DN as well as T2D + DN, indicating the regulation profile of miRNA expressions, is presented in Supplementary Figs. S2A and S2B [33]. The clustering indicated that the difference in expression levels of urinary ECVs miRNA and piRNAs between control individuals and T2D − DN as well as T2D + DN patients was the primary discriminator, overriding differences due to gender, age, and body mass index (Supplementary Fig. S3 [33]). These 25 ECVs miRNAs, which were shown to be differentially expressed between the control and T2D − DN as well as T2D + DN patients, were further analyzed using Wilcoxon rank sum tests. The results of Wilcoxon tests indicated miRNA expression in patients with T2D − DN, T2D + DN, and no T2D (healthy) (Supplementary Fig. S2B [33]). Significant change in miRNA expression was observed with 25 dysregulated in T2D − DN and T2D + DN patients compared with healthy individuals (Supplementary Fig. S2B [33]). We observed 5 miRNAs that were significantly changed in T2D − DN vs T2D + DN. A significant change in expression was observed with miR-151a-3p (0.0051), miR-182-5p (*P* = .02) in T2D + DN patients compared with T2D − DN patients (Fig. 2D and 2E). Next, by considering the urine albumin to creatinine ratio (ACR), we divided the healthy, T2D − DN, and T2D + DN cohort into 3 groups: one with healthy (ACR < 30 mg/g), microalbuminuria (30 < ACR < 300 mg/g), or macroalbuminuria/late renal disease (ACR > 300 mg/g). We observed that T2D − DN and T2D + DN patients with macroalbuminuria/renal impairment had significantly higher expression of miR-151a-3p and miR-182-5p (Fig. 2F and 2G).

### The Potential Diagnostic Value of ECVs miRNAs for T2D − DN and T2D + DN

miR-151a-5p and miR-182-5p showed an interesting expression pattern as they were downregulated in T2D − DN and upregulated in T2D + DN. To study the diagnostic accuracy of urinary extracellular vesicles miR-151a-5p and miR-182-5p as surrogate biomarkers for T2D − DN or T2D + DN, a ROC curve was drawn. The results demonstrated the diagnostic accuracy of miR-151a-5p and miR-182-5p and are shown in Fig. 2H and 2I. The AUC values indicate that individuals with differing ACR can be distinguished by expression analysis of the markers. These data indicate that miR-151a-5p and miR-182-5p miRNAs are valuable potential urinary-based miRNA biomarkers not only for T2D + DN but also for detecting future renal impairment in T2D − DN patients.

**Figure 3.**
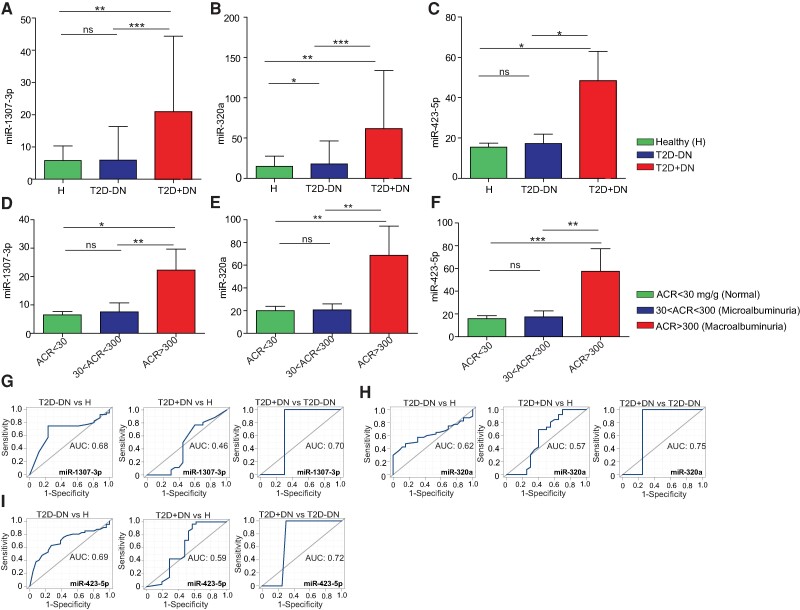
Expression profile of differentially expressed miRNAs among T2D − DN and T2D + DN. (A-C) Expression of differentially expressed miRNAs miR-1307, miR-320a, and miR-423-5p by signal values between healthy, type 2 diabetes without nephropathy (T2D − DN) and type 2 diabetes with nephropathy (T2D + DN) patients. (D-F) Box plot shows the expression of differentially expressed miRNAs (miR-1307, miR-320a, and miR-423-5p) by signal values between T2D + DN and T2D − DN patients with normal individual (ACR < 30 mg/g), microalbuminuria (30 < ACR < 300), and macroalbuminuria (ACR > 300 mg/g). (G-I) ROC curves for miR-1307, miR-320a, and miR-423-5p of T2D − DN vs heathy, T2D + DN vs healthy, and T2D + DN vs T2D − DN. The area under the ROC curve (AUC) represents the accuracy of the miR-1307, miR-320a, and miR-423-5p biomarkers in T2D − DN and T2D + DN. *indicates *P* < .05, **indicates *P* < .01, ***indicates *P* < .001 and “ns” indicates not significant.

A significant change in expression was also observed with miR-1307 (*P* = .00043), miR-320a (*P* = .002), and miR-423-5p (*P* = .0093) in T2D + DN patients compared to T2D − DN patients (Fig. 3A-3C). Significant miRNAs expression differences were seen in T2D + DN patients compared to healthy individuals and are shown in Supplementary Fig. S4 [33]. We also observed that T2D − DN and T2D + DN patients with macroalbuminuria/renal impairment had significantly higher expression of miR-1307-3p, miR-320a, and miR-423-5p (Fig. 3D-3F).

To study the diagnostic accuracy of urinary ECV miRNAs (miR-1307-3p, miR-320a, and miR-423-5p) as a surrogate biomarker for T2D − DN or T2D + DN, a ROC curve was shown in Fig. 3G-3I. The results demonstrate the diagnostic accuracy of miR-1307-3p (Fig. 3G). Similarly, miR-320a-5 and miR-423-5p performance is shown in Fig. 3H, and 3I. Importantly, significant AUC of miR-423 exhibited higher values in progression T2D − DN from T2D + DN. Compared with T2D + DN vs T2D − DN, miR-423-5p with AUC values of 0.72 had the largest area under the ROC curve with the highest sensitivity and specificity compared with miR-1307-3p (AUC = 0.7) and also miR-320a (AUC = 0.7) (Fig. 3H and 3I). The AUC values indicate that individuals with differing ACR can be distinguished by expression analysis of the markers. These data indicate that these miRNAs are valuable potential urinary-based miRNA biomarkers not only for T2D − DN and T2D + DN but also for detecting future renal impairment in T2D − DN patients.

### Prediction of Target Genes of ECV miRNAs, Functions, and Pathways of Differentially Expressed miRNAs in T2D − DN and T2D + DN

**Figure 4.**
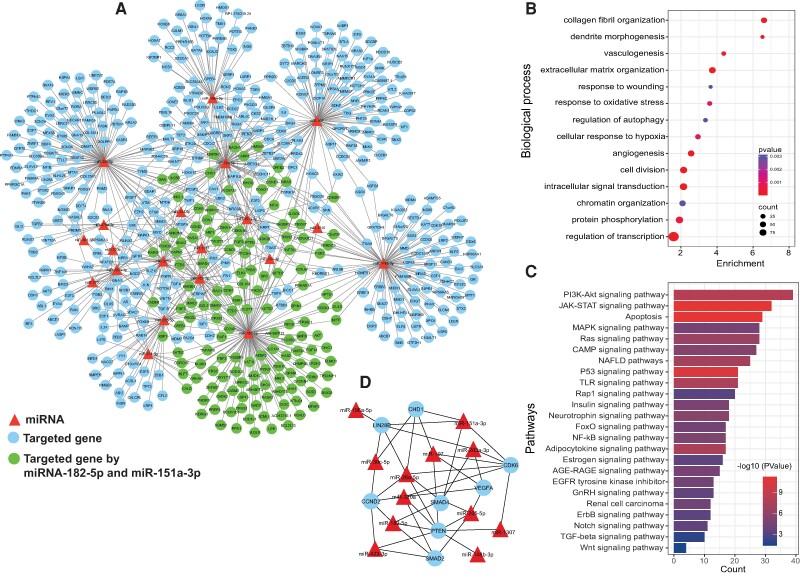
Prediction of target genes of ECV miRNAs, functions, and pathways of differentially expressed miRNAs. (A) mRNA-miRNA target interaction network obtained from a list of shared DE-miRNAs among type 2 diabetes with nephropathy (T2D + DN) and type 2 diabetes without nephropathy (T2D − DN). The network contains the significant T2D + DN and T2D − DN related miRNA-mRNA target interactions network retrieved from MIENTURNET. The miRNA is represented by red triangular shape nodes and mRNA by cyan color circular shape nodes; the green color nodes are genes targeted by prognostic miR (miR-151a and miR-182-5p) (B) Significant enriched biological process of shared DE-miRNA in T2D + DN and T2D − DN. Dot size indicates count. Count represents the number of genes associated with each pathway. Dot color denotes the *P* values of pathways and x-axis represent fold enrichment. (C) Bar plot of significant biological pathways of predicted genes of shared DE-miRNA in T2D + DN and T2D − DN patients. X-axes represent count of genes with significance (*P* < .05) indicated by order and color trend. (D) mRNA-miRNA core network module of driver genes and key DE-miRNAs.

miRNAs exert a pivotal influence on cellular mRNA expression by selectively binding to target mRNAs. This interaction results in translational repression and subsequent gene silencing. The detected 178 DE-miRNAs (including the identified 25 shared dysregulated miRNAs in urinary ECV from T2D − DN and T2D + DN patients) were found to map to 387 target genes in the miRNet database (https://www.mirnet.ca/) (Supplementary Table S5 [34]). The protein-protein interaction network built using differentially expressed miRNA-mRNA pairs revealed that the interaction network comprises 527 interacting nodes and 659 edges (Fig. 4A). We performed functional enrichment analysis of the predicted target genes from key DE-miRNA analysis. Biological pathways and KEGG pathway enrichment analysis were successively identified by DAVID (https://david.ncifcrf.gov/tools.jsp) to validate that these miRNAs are involved in T2D − DN and T2D + DN. The functional enrichment analysis indicated that the miRNA targeted genes were enriched in biological processes related to angiogenesis, response to oxidative stress (ROS), and cell division etc. (Fig. 4B). Enrichment pathways analysis revealed that the targeted genes extracellular vesicles miRNAs were enriched in Foxo, Wnt, PI3K-Akt signaling, apoptosis, MAPK signaling, p53 signaling, AGE-RAGE, JAK-STAT, insulin signaling pathways (Fig. 4C). Of interest, the top diabetes nephropathy-associated pathways of most of the extracellular vesicles key miRNAs families were related to Wnt signaling, mTOR, ErbB signaling, PI3K-Akt, and calcium signaling (Fig. 4B). In addition, more specific participation in diabetes nephropathy-related biological process analysis showed that the enriched processes were involved in cell proliferation, hypoxia, nephrogenesis, cell migration, cell differentiation, fibrosis, and DNA damage (Fig. 4C).

A protein-protein interaction network of mRNA-miRNA was constructed based on the miRNet (https://www.mirnet.ca/) database (Fig. 4A). Then, edge percolated component (EPC) algorithms in Cytoscape-cytoHubba plug-in were used to screen multicentric mRNA-miRNA based on the protein-protein interaction network. The EPC score of driver genes is given in Supplementary Table S6 [34]. Eight driver genes were identified: *PTEN, CDK6, SMAD4, CCND2, LIN28B, CHD1, VEGFA,* and *SMAD2*. The genes are listed according to EPC score in descending order (Fig. 4D).

## Discussion

To our knowledge, this study represents the first global analysis of urinary extracellular vesicle miRNAs and piRNAs—in patients with T2D and DN. By adopting a thorough approach, we were able to extensively catalog small RNAs in patient urinary ECVs using next-generation sequencing technology. We investigated the altered miRNAs and piRNAs to assess their viability as markers for DN progression. In our quest to pinpoint interaction networks and the primary target genes of significantly altered miRNAs, we relied on both theoretically derived and experimentally confirmed databases. This research offers profound insights into the mechanisms underpinning T2D and DN and identifies potential avenues for therapeutic intervention. In summary, our findings illuminate the influential role of miRNAs in the context of T2D and DN, emphasizing their importance as promising disease biomarkers and therapeutic targets.

Diabetic nephropathy is a complication that develops in approximately 40% of people with diabetes and is regarded as the leading cause of ESRD [35, 36]. However, the progression from DN to ESRD among diabetic patients varies significantly. Consequently, there is a critical need for biomarkers that can facilitate early diagnosis of the disease and identify those patients at a higher risk of rapid progression, as it would enable timely intervention leading to better outcomes.

In our study, we identified a total of 41 small RNAs, including 25 miRNAs, that are significantly dysregulated in T2D ± DN patients when compared to nondiabetic individuals. When the dysregulated miRNAs were compared across the T2D groups both with DN or without DN, 5 miRNAs were differentially expressed, including miR-1307 and miR-320a, which were previously implicated in DN [37], with the latter showing a driving role in renal dysfunction in DN patients [38]. Another microRNA was miR-423-5p, which was significantly elevated in DN patients compared to other groups. Downregulation of miR-423-5p was implicated in DN disease severity and progression using an in vitro DN animal model [39]. The other 2 miRNAs (miR-151a-3p and miR-182-5p) showed a distinctive expression pattern that might provide potential prognostic insight for patients with T2D developing kidney complications. Both markers were downregulated in the T2D − DN group and upregulated in the T2D + DN group when compared to the control group (Fig. 2). The marker miR-151a-3p has been shown previously to be downregulated in the serum of patients with lupus nephritis, which correlated with reduced renal tissue activity [40]. On the other hand, miR-182-5p was shown to be elevated following renal injury [41]. This role was further validated as in vivo inhibition of miR-182-5p improved kidney function following acute kidney injury [42].

**Figure 5.**
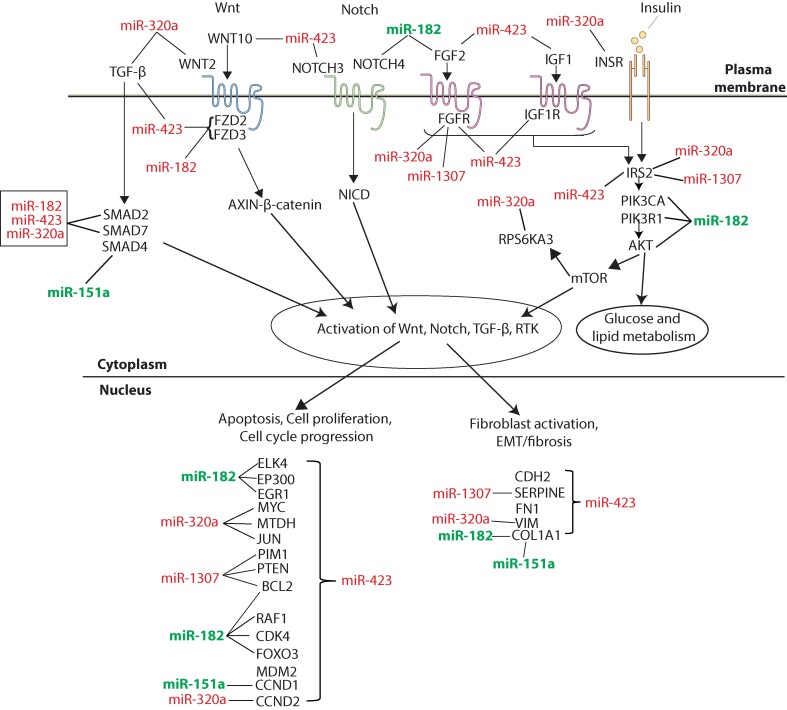
Schematic summary of the aberrant activation of transforming growth factor [TGF-β], Wnt/Noth, fibroblast, insulin growth factor, and insulin signaling pathways, and glucose and lipid metabolism in diabetes nephropathy predicted to be affected by key miRNA (miR-182, miR-151a, miR-1307, miR-320a, and miR-423) family members. Disease potential biomarkers (miR-182, miR-151a) are denoted by green color and diagnostic biomarkers (miR-1307, miR-320a, and miR-423) denoted by red color.

A summarized depiction of the aberrant activation of pathways (including growth factors/receptor tyrosine kinases [RTKs], Notch, Wnt/β-catenin, transforming growth factor-beta [TGF-β] signaling pathways, as well as glucose and lipid metabolism) in T2D with or without nephropathy is anticipated to be influenced by dysregulated key miRNAs (miR-1307, miR-320a, miR-151a, miR-182-5p, and miR-423) (Fig. 5). Regulatory analysis of significantly key dysregulated miRNA highlighted driver genes, *PTEN, SMAD2, SMAD4, VEGFA, CCND2, CDK6, LIN28B,* and *CHD1*, as well as pathways that could provide potential targets for therapeutic approaches. Among these, *PTEN* emerged as a hub affected by 8 key dysregulated miRNAs. This tumor suppressor gene has been extensively demonstrated to be involved in DN by numerous studies [43, 44], exerting its effects via the PI3K/Akt signaling pathway [45], which we identified as the pathway with the highest enrichment. *PTEN* plays an important role in regulating insulin signaling which is affected under conditions of insulin resistance [46]. PTEN has also been shown to be dysregulated in people with DN. Our findings further corroborate these findings and highlight the role of miRNAs in its regulation, allowing for possible ways for regulating *PTEN* as a promising therapeutic target for T2D and DN. Other genes regulated by the identified miRNAs are *SMAD2* and *SMAD4* that have been shown to play a critical role in DN progression via the TGF-β/SMAD signaling pathway [47].

Other genes that have been linked to the miRNAs identified in this analysis are *VEGFA* which have been shown to play an important role in DN through its role in renal angiogenesis [48]. The key hallmarks of T2D and DN are conditions of high glucose, high blood pressure, and oxidative stress that leads to disruption of angiogenesis. The kidney is a highly vascularized organ with high blood flow that requires an extensive network of blood vessels that is maintained through angiogenesis where *VEGFA* is a key regulator of this process [49]. A major problem in early stages of DN is the microangiopathy that leads to excessive abnormal angiogenesis which produces structurally undeveloped vessels which are highly pervious leading to proteinuria and increased filtration rate in the initial stages of DN due to the increased glomerular filtration surface [50]. VEGFA expression in podocytes is upregulated by high glucose after which the proteins pass through the glomerular filtration barrier and binds to VEGFR2 activating the downstream signal which leads to activation of angiogenic pathways and the dysfunction of glomerular endothelial cells in DN patients [51, 52]. Identifying urinary ECVs that regulate VEGFA further validates our approach's utility and ability to identify DN biomarkers and potential drug therapeutic targets. The involvement of *CDK6* in DN has been suggested by experimental disease models, where modulation of *CDK6* expression has been shown to mitigate various detrimental aspects of DN [53]. *LIN28B* was also shown to be involved in renal fibrosis in DN [54]. Our findings further support the crucial roles that specific pathways, and most driver genes, play in DN, suggesting that these pathways and genes may serve as potential therapeutic targets for DN in the future. In addition to these key driver genes, our analysis has also identified *CCND2* and *CHD1*, which are known to be involved in various types of cancer but have not previously been linked to DN or T2D. This leads us to propose them as novel targets for further study in DN. Identifying key miRNAs previously identified in people with DN and their link to key genes involved in DN pathogenesis, such as *PTEN* and *VEGFA*, highlights the strength of this current analysis and approach.

Determining whether the changes in small RNA profiles seen in DN are due to the disease or simply reduced eGFR is critical. Including a control group with low eGFR but no diabetes could provide clear insights. In a recent study, we demonstrated that miRNA profiles in patients with autosomal dominant polycystic kidney disease with similar eGFR reductions exhibit distinct patterns compared to our previous findings in DN [21]. These differences suggest that DN effects likely stem from its unique pathophysiology, not merely kidney function decline.

Given the scope and setup of our study, it is prudent to acknowledge the limitations in definitively answering whether the identified miRNAs are superior to ACR alone in diagnosing DN. We think, at this stage, that miRNAs should not be viewed as a replacement for ACR but rather as a complementary diagnostic tool. The potential advantage of miRNAs lies in their ability to provide additional molecular and pathophysiological insights, which could be crucial for improving the prognosis of DN and identifying new therapeutic targets. This could significantly enhance the overall management of the condition by adding a layer of molecular understanding that ACR alone may not provide.

The results presented in this study should be treated with caution due to several limitations. First, our analysis is based on a cross-sectional design, which is a significant limitation that could affect the interpretation of causality between observed biomarkers and disease progression. To further validate these findings and elucidate the potential of these miRNAs in DN and their therapeutic implications, expanding this analysis to participants enrolled in a longitudinal cohort would be beneficial. Additionally, analyzing a larger cohort would enable a more detailed examination at various stages of the disease, potentially offering deeper insights into the progression and management of DN. It is also important to note that the healthy controls were younger than the diabetic groups, which introduces an age-related variable that cannot be completely ruled out. Finally, the absence of complete medication lists for some patients restricted our ability to comprehensively assess the potential confounding effects of various drugs.

## Conclusion

Overall, miRNAs represent a promising class of biomarkers for DN that can be integrated into a multi-biomarker panel or even serve as standalone disease biomarkers. However, further research is required to ensure optimal performance. Our research findings contribute valuable insights into the understanding of DN development, revealing critical new biomarkers and pinpointing promising targets for therapeutic intervention. These discoveries might aid opening new pathways for more effective management strategies, offering hope for not only slowing the progression of DN but also for developing personalized treatment plans tailored to the specific molecular profiles of each individual’s condition.

## Data Availability

The data underlying this article will be shared upon reasonable request to the corresponding author.

## Abbreviations

ACR: urine albumin to creatinine ratio
DN: diabetic nephropathy
ECV: extracellular vesicle
eGFR: estimated glomerular filtration rate
ESRD: end-stage renal disease
miRNA: micro RNA
piRNA: Piwi-interacting RNA
T1D: type 1 diabetes
T2D: type 2 diabetes
TGF-β: transforming growth factor-beta
